# Significant influence of low positive affect on pain: impact of COVID-19 on affect and daily chronic non-cancer pain trajectories in women

**DOI:** 10.3389/fpain.2025.1612328

**Published:** 2025-11-20

**Authors:** Quinte T. Y. Kuper, Sophie F. Waardenburg, Lars Visseren, Ellen M. M. Jongen, Richel Lousberg, Therese A. M. J. van Amelsvoort, Andrea J. R. Balthasar

**Affiliations:** 1 School for Mental Health and Neuroscience, Maastricht, Netherlands; 2 Department of Anesthesiology and Pain Medicine, Maastricht University Medical Center+, Maastricht, Netherlands; 3 Department of Clinical Epidemiology and Medical Technology Assessment, Care and Public Health Research Institute (CAPHRI), Maastricht University Medical Center+, Maastricht, Netherlands; 4 Department of General Medicine, Maastricht University, Maastricht, Netherlands; 5 Faculty of Psychology, Open University,Heerlen, Netherlands; 6 Department of Psychiatry and Psychology, Maastricht University Medical Center+, Maastricht, Netherlands

**Keywords:** affect, chronic non-cancer pain, COVID-19, low positive affect, experience sampling method

## Abstract

**Objective:**

Chronic non-cancer pain (CNCP) affects 12% of the Dutch population, with similar rates in other Western countries. CNCP not only influences the physical aspects of the body but also has a relationship with affect. Affect can be positive (PA) or negative (NA). This study investigated the relationship between pain and affect and how this relationship may have differed before and during the coronavirus disease 2019 (COVID-19) pandemic.

**Methods:**

In this prospective study, patients were recruited during a standard pre-consultation visit at an outpatient pain clinic. The novelty of this approach lies in the utilisation of the experience sampling method (ESM). Patients were asked to complete an ESM digital tool 10 times a day for six consecutive days. They were categorised into the pre-COVID-19 (before March 20, 2020; *n* = 14) and during-COVID-19 (after March 20, 2020; *n* = 11) groups. The study cohort consisted of females only.

**Results:**

Patient pain levels, NA, and PA were assessed. Patients with a low PA during the pandemic experienced a significant negative impact on their daily pain levels, correlating with a 2.7-point increase on a 0–10 numeric rating scale.

**Conclusions:**

Unlike the previous focus on the effect of high NA on pain, this study emphasises the negative influence of low PA, which can likely be attributed to reduced hedonic activities during global life events, such as the COVID-19 pandemic. Understanding the micro-level impact of low PA on individuals may provide novel targeted treatment approaches for chronic pain management.

## Introduction

Chronic non-cancer pain (CNCP), defined as pain not caused by cancer and persisting or recurring for at least 3 months, is a clinical phenomenon that becomes increasingly complex over time (1, 2).

In a survey conducted in the Netherlands, 25% of the population above the age of 18 experienced chronic pain, with an apparent gender gap as 31% of the women experience chronic pain, opposed to 18% of the males. Of those with chronic pain, 70% experiencing daily pain and 83% experiencing hindrance in their day-to-day activities due to pain, with 67% relying on pain medication (3). This trend is consistent with observations in other Western societies (4). Another study similarly found that women are significantly more likely than men to experience chronic non-cancer pain, reporting an odds ratio of 1.45 (5).

In addition to its somatic-physiological component, CNCP exerts a significant impact at the psychosocial level. Pain negatively affects social systems (the complex network of relationships) and influences human interactions within society (6). Affect can be categorised as positive (PA) and negative (NA). PA encompasses emotions such as cheerfulness, relaxation, enthusiasm, and satisfaction, whereas NA includes feelings of anger, anxiety, loneliness, insecurity, and irritation. PA and NA are independent of each other and can coexist (7). For example, an individual who secures a new job in a different city may experience excitement, joy, and achievement, while simultaneously feeling loneliness or anxiety due to leaving their family and friends behind. Although numerous chronic pain interventions target NA via cognitive-behavioural and mindfulness-based therapies (8–10), the potential therapeutic importance of PA remains underexplored.

Against this backdrop, the coronavirus disease 2019 (COVID-19) pandemic presented an unprecedented opportunity to investigate how CNCP evolves under drastic lifestyle alterations. Previous studies have reported that PA can be obtained through hedonic activities, which declined during the COVID-19 pandemic (11). Concurrently, there was an increase in mental health symptoms associated with NA, such as anxiety and depression (12). In the Netherlands, the implemented lockdowns led to the closure of public venues and restricted social interactions (13). Such abrupt transformations in daily routines prompt critical questions about whether and how life events might influence the interplay between affect and pain.

Evidence has been reported that there are psychosocial impacts of COVID-19 (reduced activity, social isolation, or care disruptions) in chronic pain or chronic disease cohorts (14, 15). However, relatively little is known with respect to the relationship between daily affect and CNCP in the context of these COVID-19-related disruptions.

In this study we utilised the experience sampling method (ESM), a diary-type sampling method for ‘real-time’ data collection (16), to examine whether the COVID-19 pandemic influenced the dynamics of CNCP. Specifically, we aimed to (1) compare day-to-day and within-day changes in CNCP before and during the COVID-19 pandemic; and (2) investigate the individual and combined effects of NA and PA on pain. We hypothesised that a high NA would have exacerbated pain more during the pandemic than before it, while a high PA would have mitigated pain more during the pandemic than before it.

## Method

### Study design

This prospective observational study, planned before the COVID-19 pandemic, encountered interruptions due to two subsequent lockdowns in the Netherlands (in March and November 2020), leading to premature cessation of the study in November 2020.

### Ethics

The protocol was approved by the local research ethics committee (Medisch Ethische Toetsingscommissie van het azM/MUMC+) of the Maastricht University Medical Center in Maastricht, P. Debeyelaan 25, 6202 AZ Maastricht the Netherlands, during a meeting chaired by Prof. Dr. J.G. Maessen on January 29, 2019 (METC number 2018-0955). This study was conducted in accordance with the ethical principles outlined in the Declaration of Helsinki in 1975, as revised in 1983. The study began on 07-06-2019 and ended on 10-11-2020. All included patients gave written informed consent.

### Inclusion and exclusion criteria

The inclusion criteria were: (1) online informed consent provided for the use of data in scientific research, (2) completion of a standard digital intake questionnaire at the outpatient pain clinic, (3) owns a smartphone, (4) proficiency in the Dutch language to answer the ESM questions, and (5) pain duration ≥ 3 months. The exclusion criteria were: (1) < 18 years of age and (2) cancer diagnoses.

### Data collection

Data collection utilised the Psymate app (http://www.psymate.eu) as a digital ESM tool, validated by van Os et al. (16). The Psymate app is a smartphone application for the real-time collection of data on an individual's thoughts, feelings, and activities at various intervals throughout the day (17). For six full consecutive days before their initial physician appointment, participants received 10 semi-random acoustic alerts per day, evenly distributed between 7:30 a.m. and 10:30 p.m. This scheduling ensured an even distribution of alerts throughout the day while minimising anticipation effects. Patients were briefed on the purpose of the study and were instructed to keep their smartphone sound on to avoid missing alerts. Each prompt contained the same set of 18 questions assessing current pain levels, affect, and contextual factors (Supplementary Table S1), and a report was valid if completed within 15 min of the alert. A sample size of at least 18 repeated ESM reports per patient (30% of the maximum of 60) were necessary for reasonable statistical power (17).

As part of the standard digital intake questionnaire at the MUMC + pain clinic, patients completed questionnaires assessing pain complaints, quality of life, anxiety, and depressive symptoms, including the Dutch version of the Short Form Health Survey 12 (SF-12) (18) and the Hospital Anxiety and Depression Scale (HADS) (19), prior to initiating ESM digital tool usage. The physical and mental health component scores of the SF-12 (SF-12 PCS and MCS) and the total HADS score served as covariates in the analyses. Additional information was obtained from medical records, including body mass index (BMI), primary diagnosis (categorised as lumbosacral radicular syndrome, peripheral nerve pain, or nociceptive pain), and prior pain clinic attendance (0 = no, 1 = yes). Education was dichotomised into high (bachelor's degree or higher) vs. lower levels; pain duration (in years) reflected the length of the participants’ chronic pain complaints; and pain medication use before the study (0 = no, 1 = yes) was recorded.

The Psymate app also captured two time-based variables—day number (1–6) and short report number within each day (0–10)—as well as the number of completed ESM prompts per day, allowing analysis of both between-day and intra-day fluctuations in pain and affect. Pain intensity was recorded on an 11-point numeric rating scale (NRS; from 0 = ‘no pain’ to 10 = ‘worst imaginable pain’), and affect was measured via five positive items (PA) and five negative items (NA), each rated on a 7-point scale (from 1 = ‘not at all’ to 7 = ‘very much’), consistent with the expanded version of the Positive and Negative Affect Schedule [5]. Participants were further classified according to the COVID-19 pandemic period, distinguishing those included before (0) or on/after (1) 20 March 2020—the start of the first lockdown in the Netherlands (13). Finally, a dummy variable for activity (1 = ‘high’, 0 = ‘low’) was constructed from the ESM prompts to differentiate tasks such as working, housework, sports, or caring for others (high) from resting, eating/drinking, self-care, or relaxing (low). This data collection approach is consistent with the measures described by van Os et al. (16) and in the Dutch Data Pain study (20). For a more detailed understanding of the methodology involving ESM and the questionnaires employed, readers are referred to the detailed explanation provided in Waardenburg et al. (21). All the predictor variables were based on literature and the researcher knowledge and experience. Data is available upon request to the corresponding author. The current study is compliant with the STROBE guidelines.

### Statistical analyses

Prior to conducting multilevel regression analyses, descriptive analyses were performed to gain basic insight into the distribution of variables and to better understand the characteristics of the subgroups. *T*-tests were used to assess continuous variables, while chi-square tests were used to assess categorical variables, as shown in Table 1. In cases of low cell frequencies (<5), Fisher's exact test was used.

**Table 1:** Patient characteristics before and during the COVID-19 pandemic. No significant statistical differences were found between the groups (*p* > 0.1).

Variable	Pre-COVID-19	During-COVID-19
	Frequency or mean ± standard deviation	
Age	47.93 ± 11.385 years	45.27 ± 11,288 years
BMI	26.22 ± 4.58 kg/m^2^	29.18 ± 4.87 kg/m^2^
Short reports per person	38.13	41.20
Diagnosis	Lumbosacral radicular syndrome: 4 Peripheral nerve pain: 3 Nociceptive pain: 7	Lumbosacral radicular syndrome: 2 Peripheral nerve pain: 3 Nociceptive pain: 6
Education	High: 4 Low: 10	High: 3 Low: 8
Duration of pain	2.79 ± 0.43 years	2.91 ± 0.30 years
Pain medication	Yes: 10 No: 3 Missing: 1	Yes: 7 No: 4
SF-12 score^a^	PCS: 29.23 ± 7.14 MCS: 44.62 ± 9.49	PCS: 26.10 ± 5.17 MCS: 48.20 ± 12.34
HADS-score^b^	13.64 ± 7.66	13.82 ± 7.35
*Activity (0* *=* *low, 1* *=* *high)*	0.29 ± 0.47	0.27 ± 0.47
Visited a pain clinic before	Yes: 9 No: 4 Missing: 1	Yes: 3 No: 7 Missing: 1

COVID-19, coronavirus disease 2019; BMI, body mass index; SF-12, Short-Form Health survey 12^a^, HADS, Hospital anxiety scale^b^, PCS, Physical Component Score; MCS, Mental Component Score.

Multilevel analyses were performed instead of the traditional analysis of variance and/or linear regression analysis. It is crucial to acknowledge that the consecutive short reports form a series of repeated measures which are nested within a subject. It is therefore necessary to adjust for this nesting, i.e., the dependency between subsequent measures within a subject. Multilevel analysis emerges as the preferred technique for analysing such repeated measure designs (22). Furthermore, employing multilevel analysis facilitates the incorporation of a random intercept and random slopes. Thus, as the consecutive short reports (Level 2) were nested within the patient (Level 1) cohort, a dual-level structure was applied. In all models, pain served as the dependent variable. A random intercept was included as it is highly likely that the ‘base level’ of pain differs between patients. A random slope was modelled for the variable ’short_report_occurrence_within_day’ (Supplementary Table S2) as the linear trend of is also expected to vary between patients. Supplementary Table S2 contains the multilevel regression models, encompassing five second-order and two third-order interactions. The analyses strategy was similar to earlier studies utilizing the psymate app as a digital ESM-tool (23, 24).

An analysis of the interaction between PA, NA, and time within the day was conducted separately for the pre-COVID-19 and during-COVID-19 periods. Additionally, a similar examination of the third-order interaction effects involving NA was conducted. SPSS Statistics for Windows, version 28.0 (IBM, Armonk, NY, USA), was utilised for all statistical analyses with the level of statistical significance set at *p* < 0.05.

## Results

Initially, 217 patients were approached for the study. Of these, 168 individuals declined participation, whereas 49 provided informed consent. Four of them were excluded owing to pain complaints lasting less than 3 months, and one was excluded because of missing baseline data on sex, yielding a sample of 44 chronic pain patients (13 men and 31 women). A further ten patients were excluded owing to insufficient completion of short reports (> 30%), and two were excluded for failing to attend the first physician appointment. No males were included during the COVID-19 period. Since it is known that there is a main gender effect on reported pain (our dependent variable) and coping strategies (25, 26), we therefore could not ensure comparability between both cohorts. It was therefore decided to exclude all males and perform the analysis. Subsequently, the final dataset comprised 25 female patients—patients were considered female according to their sex on their passports. Among them, 11 patients were enrolled during the coronavirus disease (COVID-19) pandemic, while 14 were included before the pandemic.

A frequency analysis confirmed the normal distribution of the pain variable (mean = 5.74, standard deviation = 2.28, skewness = −0.53, kurtosis = −0.43). No outliers were identified. Similar to previous Psymate app studies (24)., the autoregressive (AR1) covariance structure demonstrated the best fit with the data. As expected, the AR1 diagonal was highly significant (*p* < 0.001).

To assess the homogeneity of the two subgroups, we compared all predictors and patient characteristics between them (Table 1). None of these predictors differed significantly between the two groups (all *p* > 0.1). The dataset comprised 1,003 short reports, with an average of 40 (out of a possible 60) responses per patient over 6 days. Detailed patient characteristics are presented in Table 1.

### General influences of time and affect on pain

Initially, a regression model was employed to assess the prevalence of any between-day effect on the pain reports. The mean of the PA items was considerably different from the mean of the NA items (4.4 vs. 1.9). Furthermore, NA distributed skweded (skewness is 1.47 S.E.= 0.078). Therefore, to facilitate comparison, both the PA and NA sum scores were dichotomised (low = 0 vs. high = 1) using a median split. No between-day effect was observed (*p* = 0.47). Subsequently, the within-day effect, as indicated by the occurrence of short reports, was introduced as a predictor of pain. This analysis yielded a significant result (*p* < 0.001), suggesting a linear increase in pain per short report throughout the day.

In the subsequent analysis, we aimed to clarify the relationship between pain and affect. In the regression analyses both NA and PA demonstrated independent effects on pain. The estimate (coefficient) of NA was 0.295 (*p* = 0.006) while the estimate of PA was −0.373 (*p* < 0.001). As evident from the effects, PA demonstrated a decreasing effect on pain, while NA exhibited an increasing effect.

### Investigation of differential relationships between pain and affect during the pre- and during-COVID-19 periods

To explore potential variations in the relationship between pain and affect during the pre- and during-COVID-19 periods, interaction effects with PA/NA pre- and during COVID-19 (outlined in Supplementary Table S2) were incorporated into the regression model.

**Figure 1:**
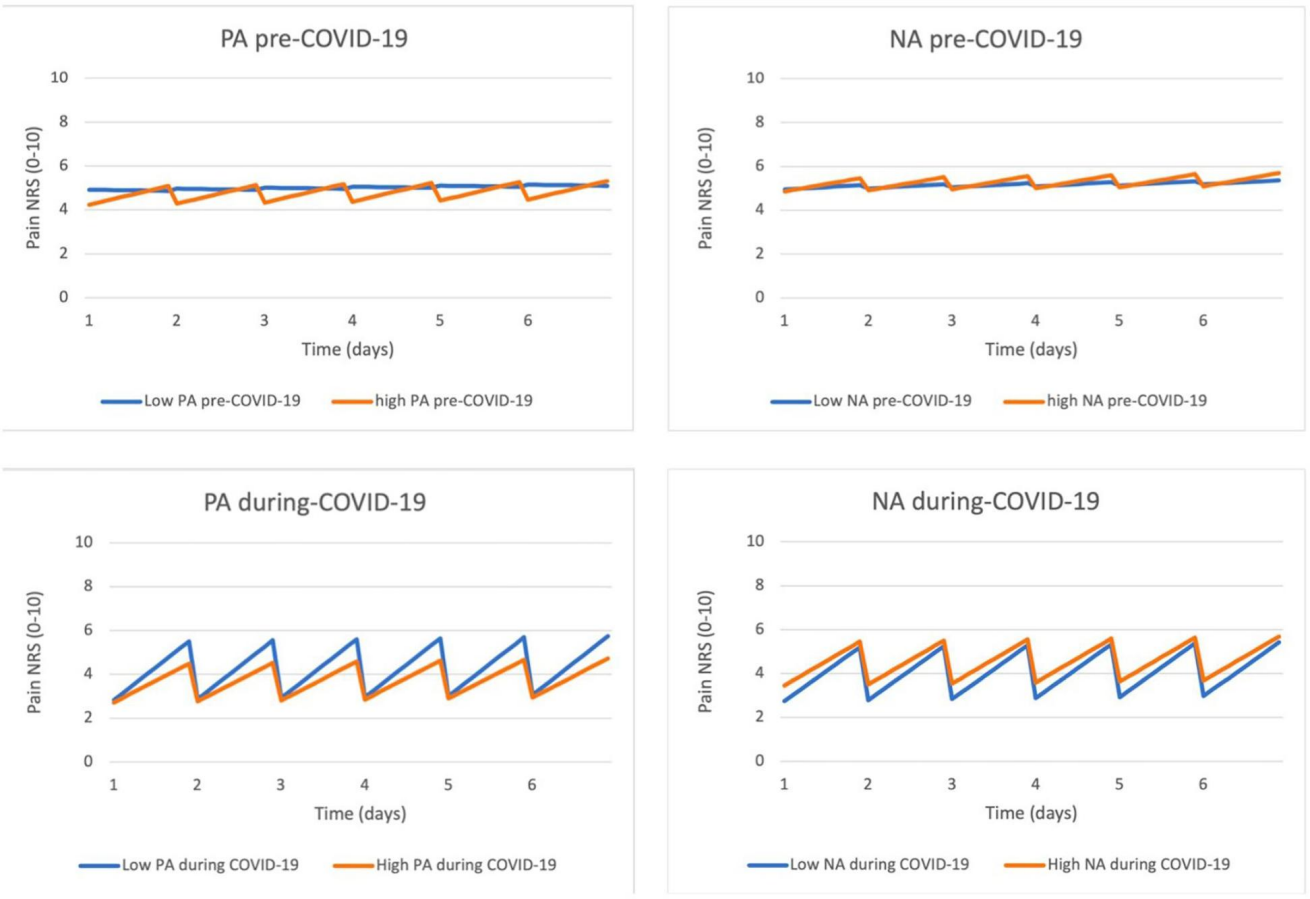
The effect of effect on chronic non-cancer pain, before and during the COVID-19 period.

Upon inspecting the pain courses depicted in Figure 1, a more pronounced increase in pain throughout the day was observed during the COVID-19 period compared to the pre-COVID-19 period. Additionally, the interaction between affect and pain seemed to differ for NA and PA. Regarding low or high NA, the increase in pain throughout 1 day during the COVID-19 period was approximately the same. In contrast, low or high PA exhibited a distinct dynamic during the COVID-19 period, with the increase in pain during the day appearing to be larger in cases of low PA.

To explore potential variations in the relationship between pain and affect in the pre- and during-COVID-19 periods, we incorporated the interaction effects outlined in Table 2 into the regression model. To visually represent the third-order interaction effects, we generated four figures (Figure 1). In these figures, the horizontal axis represents time (spanning six consecutive days) and the occurrence of short reports (indicating the within-day effect). The vertical axis predicts pain levels, ranging from 0 to 10. The two-coloured lines correspond to the interactions between the pre- and during-COVID-19 periods and high and low levels of NA and PA.

**Table 2:** Third-order interactions showing how NA and PA influenced pain before and during the COVID-19 pandemic.

3rd order interaction^a^	Coefficient	Std. Error	t-value	*p*-value	95% CI LB	95% CI UB
NA	−0.0998	0.0824	−1.185	0.237	−0.265	0.0657
PA	−0.2051	0.0775	−2.645	0.008^b^	−0.357	−0.0523

^a^Construction of the third-order interaction is shown in the SPSS syntax in Table S3.

^b^Significant interaction.

COVID-19, coronavirus disease 2019; NA, negative affect; PA, positive affect; CI, confidence interval; LB, lower border; UB, upper border.

Although the second-order interaction term, COVID-19 × time within the day, was not significant (t = 1.79, *p* = 0.09), this trend cannot be ignored. No significant difference was found in the interaction effect with NA (Table 2). This effect corresponded to a 1.8 increase in reported pain on the NRS-scale. Conversely, the third-order interaction effect with PA was significant, *p* = 0.008 (Table 2). This effect corresponded to a 2.7 increase in reported pain during low PA during the COVID-19 period.

*Post-hoc* analyses, in which all covariates were excluded, yielded the same overall results. The third-order interaction effect with NA remained non-significant (*p* = .973), whereas the third-order interaction with PA remained significant (*p* = .009), demonstrating the robustness of the data.

## Discussion and conclusions

This study examined pain experiences and their relationship with affect in Dutch women before and during the COVID-19 pandemic. ESM was used to examine whether the COVID-19 pandemic influenced the effect of affect within the day on CNCP. Specifically, we aimed to: (1) compare day-to-day and within day changes in CNCP before and during the COVID-19 pandemic; and (2) investigate the individual and combined effects of NA and PA on pain.

We hypothesised that a high NA would have exacerbated pain more during the pandemic than during the pre-pandemic period, while a high PA would have mitigated pain more during the pandemic than during the pre-pandemic period.

During the COVID−19 period, there appeared to be a marked increase in pain throughout the day compared to the pre-COVID-19 period; although this increase was not significant, it was interpreted as a possible trend. No significant differences were found in predictors between the pre- and during-COVID-19 subgroups, except for the absence of male participants. A higher NA was linked to more pain, whereas a higher PA was associated with less pain, indicating that NA and PA have opposite and independent influences on pain. The interaction between affect and pain differed during the pandemic, particularly for low PA, which was associated with a larger increase in pain. These findings highlight the complex interplay between affect and pain, especially during times of heightened stress like during the COVID-19 pandemic.

Data collection for this study commenced in 2019, predating the onset of the COVID-19 pandemic. Our primary objective was to explore pain–time effects within a cohort of patients with CNCP. Owing to the occurrence of the COVID-19 pandemic we divided the cohort into pre- and during-pandemic groups. However, no significant pain–time effects were observed between days in this CNCP cohort, consistent with the understanding that chronic pain operates differently from acute pain. Unlike acute pain that typically follows a diminishing temporal trajectory during the healing process, chronic pain tends to exhibit greater stability over time (27, 28). Furthermore, acute pain serves as a self-limiting response, acting as a protective biological mechanism during tissue healing. In contrast, chronic pain is characterised by the intricate interplay of multiple factors, leading to a constellation of symptoms that resist resolution through conventional medical approaches (29). The absence of a temporal effect in our chronic pain population underscores the chronic character of the pain of these patients.

Upon closer examination of the temporal aspects of pain, we identified a significant within-day effect, which became apparent through the ten short reports recorded each day. This finding highlights the susceptibility of pain to external influences (30). The intermittent patient reports collected throughout the day offer useful insights into the dynamic and fluctuating nature of pain, revealing intricate links to various external stimuli and conditions. The recognition of within-day variations of NA and PA contributes to a more nuanced understanding of the intricate interplay between affect, pain perception, and external factors such as relationships, work, and environment.

As a second objective, we explored the overall relationship between pain and affect. Both NA and PA demonstrated a significant association with pain, aligning with clinical experience and previous studies indicating a connection between affective states and temporal pain (31). This relationship was also observed in a study involving patients with acute pain and experience sampling use (32).

We explored the pandemic's impact on the CNCP cohort, offering insight into the influence of major global events on the relationship between emotions and pain. Categorising affect as PA or NA, we regarded both as separate pain effects. During the pandemic, patients with high NA experienced exacerbated pain during the day compared to pre-pandemic patients with CNPC, consistent with previous findings linking high NA to increased pain (24–30). Another study suggested that life events, including emotions associated with NA, directly influence pain (33).

Patients experiencing low PA during the COVID-19 pandemic had a worsened pain trajectory during the day compared to those before the pandemic. Distinguishing between low PA and high NA is crucial. The former indicates a deficit in positive emotions, while the latter implies an abundance of negative emotions. Despite research exploring the influence of high PA during major life events, the consequences of low PA during such occurrences remain less studied, highlighting a notable gap in current understanding (9).

Especially, the early periods of COVID-19 pandemic itself can be seen as a major life event. It is a period of elevated psychosocial stress, disruption of daily lives, limited access to care, and increased emotional burden. This is particularly relevant for women, who are more likely to experience a reduction in hedonic activities—such as socializing, hobbies, or physical activity—that typically promote positive emotions and well-being (34). These disruptions could have contributed to altered affective patterns and heightened pain sensitivity, therefore making the pandemic an important setting to study the relationship between affect and CNCP.

The finding that a low PA had a negative impact on pain in women during the COVID-19 pandemic suggests that a low PA holds greater clinical relevance than a high NA in patients with CNCP. Patients with a low PA exhibited a significant increase of 2.7 points on the 11-point NRS during the day. However, those with a high NA showed a smaller, non-significant but clinically relevant, increase of 1.8 points on the same scale. The observed pain elevations, both clinically relevant, align with established criteria defining a meaningful change in pain level. Previous studies have indicated that an elevation in pain of at least 20% between two time points, equivalent to a score of 1.8 on the 11-point NRS, is considered clinically significant (35).

This contrast underscores the pivotal role of low PA in shaping daily pain experiences in women during the COVID-19 pandemic. It suggests the need for a shift in therapeutic focus from high NA to low PA in CNCP treatment.

Analysis of identified relationships revealed that both NA and PA exhibited less favourable trajectories in daily pain experiences during COVID-19, regardless of initial affect levels. This differed from the observations of the pre-COVID-19 period. This pattern suggests the presence of a general COVID-19 effect on daily pain trajectory, highlighting the influential role of life events in shaping daily pain experiences. It is crucial to contextualise the during-COVID-19 data temporally, as it was collected between the initial and subsequent lockdowns in the Netherlands. The more pronounced impact of COVID-19 during these early lockdowns and surges may have contributed significantly to the observed effects, emphasising the dynamic relationship between life events and pain experiences. The results contrast with our hypothesis; we expected to find a more profound role of NA in CNCP. Instead, we found that low PA seems to have a more profound role in CNCP.

These findings suggest the need for healthcare professionals and researchers to reconsider and enrich their therapeutic approaches, especially considering the emotional dimensions experienced by individuals with low PA during significant life events. Recognising the profound influence of low PA on pain outcomes opens opportunities for developing tailored healthcare interventions. Such personalised approaches could lead to more effective strategies for managing and alleviating CNCP, particularly during life events. Positive psychological interventions, which have shown efficacy in enhancing PA, may play a pivotal role in improving chronic pain management outcomes (36, 37).

The intricate relationship between pain and emotion is potentially rooted in shared neural mechanisms, highlighting the interconnected nature of these experiences. The significant overlap between the neural mechanisms of pain and emotion, particularly in shared regions like the insula and cingulate cortices, underscores their convergence in interoceptive processing. This interplay is crucial for shaping the brain's representation of the body's internal state (31). While the effects of affect on pain vary considerably between individuals, the intrinsic link between the two emphasises the need to address emotional dimensions as part of comprehensive pain treatment. Furthermore, acknowledging individual variability in responses to interventions is essential for optimising CNCP management (30).

Despite its strengths, this study has some limitations. First, of all individuals approached, 77% declined participation. This high refusal rate can likely be explained by the intensive nature of the study. Participants were asked to complete multiple short reports per day over six consecutive days. Given this demanding schedule, it is understandable that many declined to participate. Consequently, those who agreed to take part may represent a subgroup that is more motivated or better able to engage with intensive research protocols, which may have introduced a selection bias. Second, as this study was conducted partly during the COVID-19 pandemic, the sample size was limited, which may have reduced the reliability of the findings, and it can therefore only be seen as exploratory. However, despite this challenge, we utilised ESM for data collection, obtaining data 10 times a day over six consecutive days. This extensive data collection resulted in a substantial dataset and statistical power for meaningful analyses between the two sub-cohorts. Additionally, due to the application of ESM, recall bias was avoided. Notably, the during-COVID-19 group comprised only female participants, limiting gender representativeness. Therefore, all male participants were excluded. This limits gender representation and introduces a sex bias.

In addition, we included only participants who completed a minimum of 30% of the short reports, leading to a selection bias of 22%. This potential source of bias may affect the generalisability of our findings.

Despite these limitations, the dataset is unique in that it was obtained before and during the COVID-19 pandemic. Owing to the unique circumstances surrounding this pandemic, the findings are not easily replicable. Nevertheless, the extensive data collection, high mean short report response rate (66.6%), and strong statistical power within the patient data (1003 short reports) support the robustness of the study's dataset.

Considering these findings, healthcare professionals are invited to carefully re-evaluate therapeutic approaches, particularly focusing on the emotional dimensions of patients experiencing low PA during significant life events. This study highlights the pivotal role of low PA in shaping daily pain experiences, emphasising the need to include and shift the therapeutic focus from solely addressing high NA to also addressing low PA in CNCP treatment. Positive psychology interventions could play a crucial role in this shift. Future research should aim to overcome these limitations by expanding sample sizes, addressing biases, and ensuring sex representativeness in participant pools, thereby fostering a more comprehensive understanding of the complex relationship between affect and chronic pain during life events.

## Data Availability

The raw data supporting the conclusions of this article is available upon request, without undue reservation.
